# Are Retinal Vessels Calibers Influenced by Blood Pressure Measured at the Time of Retinography Acquisition?

**DOI:** 10.1371/journal.pone.0136678

**Published:** 2015-09-16

**Authors:** Sandra C. Fuchs, Helena M. Pakter, Marcelo K. Maestri, Marina Beltrami-Moreira, Miguel Gus, Leila B. Moreira, Manuel M. Oliveira, Flavio D. Fuchs

**Affiliations:** 1 Postgraduate Studies Program in Epidemiology, School of Medicine, Universidade Federal do Rio Grande do Sul, R. Ramiro Barcelos 2600, CEP 90035–003, Porto Alegre, RS, Brazil; 2 Postgraduate Studies Program in Cardiology, School of Medicine, and Hospital de Clinicas de Porto Alegre (HCPA), Universidade Federal do Rio Grande do Sul, R. Ramiro Barcelos 2600, CEP 90035–003, Porto Alegre, RS, Brazil; 3 Division of Ophthalmology, Hospital de Clinicas de Porto Alegre, Universidade Federal do Rio Grande do Sul, R. Ramiro Barcelos 2350, CEP 90035–003, Porto Alegre, RS, Brazil; 4 Informatics Institute, Universidade Federal do Rio Grande do Sul, Caixa Postal 15064, CEP 91501–970, Porto Alegre, RS, Brazil; 5 National Institute of Science and Technology for Health Technology Assessment (IATS)-CNPq, Hospital de Clinicas de Porto Alegre, UFRGS, Porto Alegre, Brazil; Osaka University Graduate School of Medicine, JAPAN

## Abstract

**Background:**

Retinal arterial narrowing is associated with higher office blood pressure (BP) and ambulatory blood pressure monitoring, and increased incidence of cardiovascular disease, but it is still unknown if the vessel caliber is associated with BP measured at the time of retinography acquisition.

**Methods:**

Retinal arteriolar and venular calibers were measured by the microdensitometric method in 448 patients with hypertension. Participants underwent 24-hours ambulatory blood pressure (24-h ABP) monitoring simultaneously with the retinography acquisition. Association between arteriolar and venular calibers with increase of 10 mmHg in the mean 24-hours, daily, and nightly BP, and with BP measured at the time of retinography, was evaluated by ANOVA and multivariate analyses.

**Results:**

Mean 24-hours, daytime and nighttime systolic and diastolic BP were inversely associated with the arteriolar caliber, but not with the venular caliber. Arteriolar caliber decreased -0.8 (95% CI -1.4 to -0.2) μm per 10-mmHg increase in 24-hours mean systolic BP, adjusted for age, gender, fellow vessel, and duration of hypertension (P = 0.01). The corresponding decreasing in arteriolar caliber by 10 mmHg of increasing in mean diastolic BP was -1.1 μm (-2.0 to -0.2, P = 0.02). The decrease of arteriolar caliber by the same increasing of BP measured at the time of retinography was lower and not statistically significant, particularly for mean diastolic BP and outer arterioles calibers: -1.0 (-1.8 to -0.2) μm in the daytime BP average versus -0.3 (-0.9 to 0.3) at the moment of retinography acquisition.

**Conclusions:**

These findings suggest that the caliber of arteriolar retinal vessels in patients with uncontrolled hypertension are not significantly influenced by blood pressure measured at the time of retinography acquisition.

## Introduction

The classical studies linking abnormalities in retinal vessels diameters and hypertension were based on direct examination of optic fundi and in office blood pressure measurement [1]. The direct observation of retinal vessels was open to measurement bias [2] and new methods of digital image processing of retinographies have been developed [3–8] to improve the measurements of retinal vessels diameters and their association with cardiovascular disease [8,9]. Narrowing of the retinal vessel lumen [10] has been associated with higher incidence of cardiovascular events independently of blood pressure [11,12], and may even occur previously to the incidence of clinical hypertension [12].

Blood pressure has been measured only at the office and not by 24-hour ambulatory blood pressure (24-h ABP) monitoring in epidemiological studies [11,12]. Overall, studies with blood pressure assessed by 24-h ABP monitoring have been more precise to predict cardiovascular events [13,14]. Studies looking at the association between blood pressure and optic fundi abnormalities did not examine BP and retinal vessels at the same time. We demonstrated that microdensitometric method measures the column of blood in the retinal arterioles [10]. Decreasing of the caliber vessel could be therefore caused by thickening of the arteriolar wall or vessel constriction. It is unclear if the caliber of retinal vessels is caused only by histological abnormalities of vessel walls or if the diameter can be influenced by the degree of vasoconstriction/vasodilation secondary do BP levels at the moment of examination. The aims of our study was to investigate the association of retinal vessel calibers with the mean 24-h ABP measured at the time of the retinography acquisition to explore if there is an instantaneous association between blood pressure levels and retinal vessels calibers.

## Material and Methods

### Study population

This cross-sectional study was done with patients screened to participate in the MONITOR study (Randomized clinical trial of efficacy of home blood pressure monitoring and target organ damage; ClinicalTrials.gov NCT00921791) [15]. This study was conducted in the Hypertension Clinic of the Division of Cardiology of Hospital de Clínicas de Porto Alegre (Porto Alegre, Brazil). Patients aged 18 to 80 years, referred with a medical diagnosis of hypertension, were eligible. The exclusion criteria included severe hypertension (office BP ≥ 180/110 mmHg), secondary hypertension, disabling chronic disease (cancer, liver cirrhosis, heart failure, unstable angina pectoris, mental disorders), and myocardial infarction or stroke within six months of enrollment. The results of the main study were published elsewhere [15].

#### Assessment of blood pressure

All individuals were submitted to 24-h ABP monitoring using the equipment Spacelabs 90207 (Spacelabs, Redmond, WA). Readings were obtained automatically at intervals of 15 minutes during the day (07:00 AM to •23:00 PM) and 20 minutes at night (23:00 PM to 7:00 AM). Patients with less than eight measurements during the night or 16 during the day were excluded from further analysis [16]. Mean 24-hour, daytime and nighttime systolic (SBP) and diastolic (DBP) were calculated and blood pressure measured just before retinography acquisition was specifically recorded.

#### Retinal photography and measurement of retinal vascular caliber

Fundus photography was acquired after pharmacologic pupil dilation (20 min after instillation of tropicamine) using Topcon TVR-50 retinal camera (Topcon, Japan) in a 35- degree angle centered on the optic disc. The color slides were digitized in a 35-mm film scanner Hewlett Packard model Photo Smart 20S (Hewlett Packard, Andover, MA) with a resolution of 600 dots per inch. Images were stored in 24 bits (true color). Retinography was obtained during the day of ABP monitoring, usually between 11 AM and 2 PM. The capture of retinal image and blood pressure measurement were synchronized. The picture was taken just after a measurement of blood pressure by the device.

**Fig 1:**
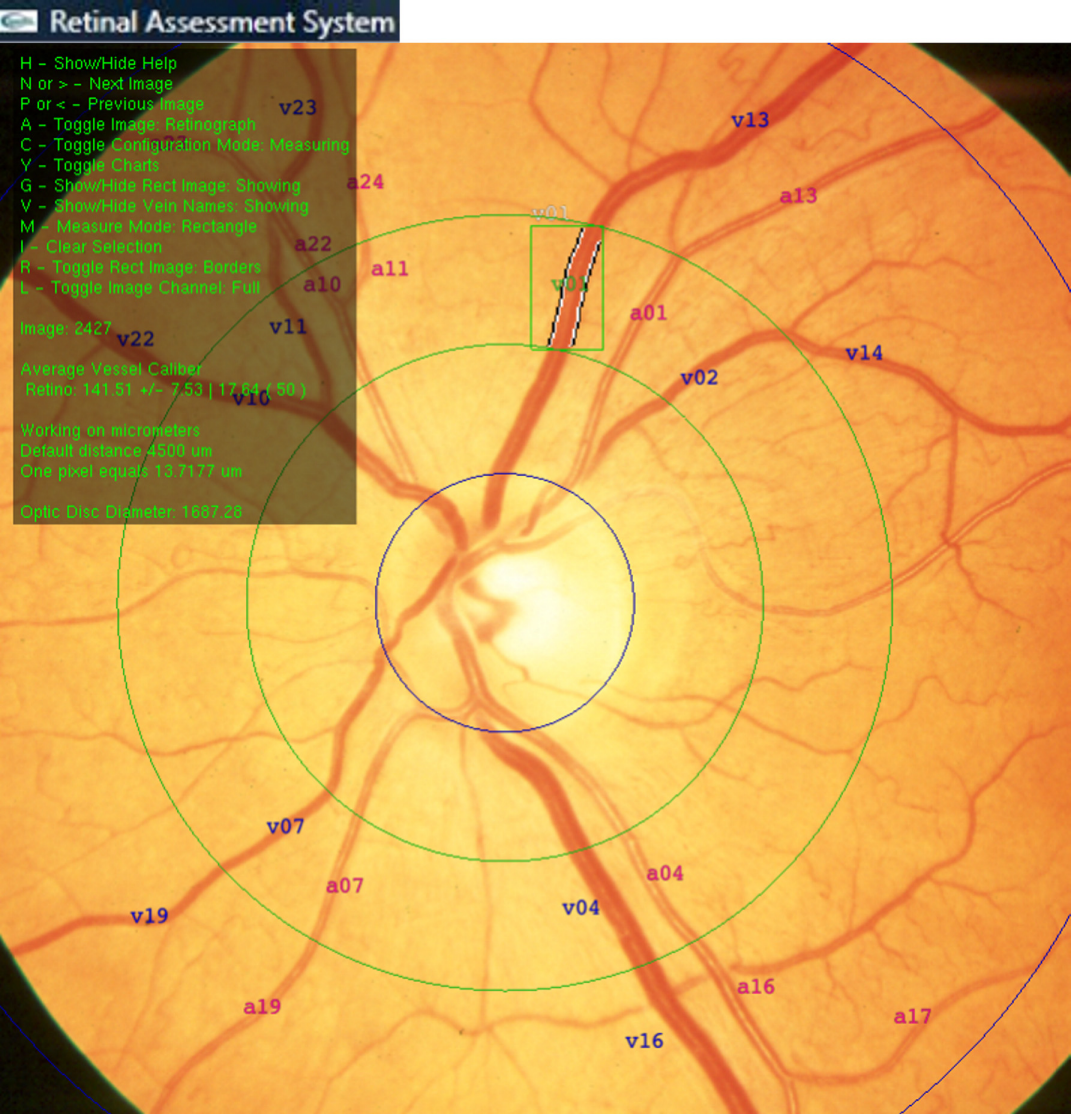
Retinal Assessment System used to determine vessel caliber.

Retinal vascular caliber was measured using an updated version of a semiautomatic computer-based program developed in our service (Retinal Assessment System, RAS, Universidade Federal do Rio Grande do Sul—Brazil) [3]. Briefly, at the opening of an image in the software, four concentric rings were automatically dropped over it. The inner circle measured the equivalent of 1800 μm (using the calibration factor of 13.72 μm/mm), and it was centered at the optic disc head. The second, third, and fourth rings have one and a half, two, and three disc diameters, respectively. If the program aligned the rings incorrectly at the optic disc, the operator adjusted them. Two concentric zones were delimited: the inner zone (A), ranging from one half of the disc diameter to one full disc diameter; and the outer zone (B), between one and two disc diameters from the margin (Fig 1) [3]. Arterioles and venulas were marked 1 to 12 in the inner zone, and 13 to 24 in the outer zone. The vessels were also marked as pairs, according to proximity and bifurcation level, to calculate arteriolar to venular ratio (AVR). Besides the traditional method of retinal assessment [17], we developed and validated a method for assessment of A/V ratio using a semiautomatic method that allows the measurement of blood column [10]. The operator identified the most visible pair of arterioles and venulas to be automatically measured in the inner and outer zones, and the distribution of pairs in each quadrant in the photograph was averaged by the software. For each measurement, a working area was constructed around the vessel of interest after setting an axis along its direction. Width measurements were performed in a plane perpendicular to this axis. For each pair of adjacent vessels, in the perpendicular direction of the wall, the operator selected a rectangular region of interest around each vessel, and the software computed the vessel boundaries (lumen) using the green channel of the retinographies. An edge detection procedure was applied to the selected image. A double convolution was done using an approximation of the Sobel operator. Sub-pixel resolution was obtained via cubic spline fitting of the edge of vessel walls. For each pair of vessels, calibers were measured and the average of approximately 94 measurements per vessel were calculated.

Measurements of vessel calibers were performed in one eye, usually the right eye. When the right eye was not feasible, the assessment was carried out in the left eye. Previously to the study, we assessed differences among eyes by measuring the scale factor in both eyes and the mean scale factor was included as a constant in the software. The scale factor was determined measuring the distance between the center of macula to center of optic disc in a sample of digitized photographs containing the macula and the optic disc in the same field. Differences between right and left eyes among the same subjects were not clinically relevant or statistically significant and gave support for the use of a single eye vessel measurement in larger studies. In addition, we tested the association of blood pressure with vessel calibers estimated by the Hubbard-Parr method [18] revised by Knudtson et al. [19].

### Sample size and statistical analysis

All analyses were performed using SPSS (Statistical Program for Social Sciences, version1 17.0: SPSS Inc., Chicago, IL). One-way ANOVA and linear regression models were used to explore the association between blood pressure and retinal vascular caliber, adjusting for age, gender, and duration of hypertension. We also controlled for anatomy and measurement errors, including the caliber of the vessel correlated in the analysis. This allows to take into account that arteriolar and venular calibers are correlated and people with narrower arterioles are more likely to have narrower venules. Systolic and diastolic blood pressures included in the analyses were the average of 24 hour, daytime, nighttime, and blood pressure measured at the time of retinography acquisition. Vascular calibers were summarized as mean arteriolar caliber, mean venular caliber, and mean AVR, for inner and outer zones, respectively, and were compared by 10 mmHg of incremental in blood pressure.

Reproducibility of the microdensitometric method was tested by an ophthalmologist who assessed the same photographs twice and by an independent ophthalmologist who verified a subsample of 20% of the photos, to determine reliability intra and inter observer, respectively [10] Bland-Altman plots and intra-class correlation coefficients were used in the analysis [20].

### Ethics statement

Patients provided free and written informed consent. The study protocol conforms to the ethical guidelines of the 1975 Declaration of Helsinki as reflected in a *priori* approval by the institution's human research committee of our Institution (Hospital de Clinicas de Porto Alegre, number of registration: GPPG: no.04-465), which is accredited by the US Office of Human Research Protections as an Institutional Review Board.

## Results

Table 1 shows the characteristics of the study population, which are typical of an outpatient clinic of hypertension. Patients were using on average of 1.4 ±1.2 anti-hypertensive drugs, mostly ACE inhibitors (47%), diuretics (34%), β-blockers (34%), and amlodipine (14%). A total of 560 individuals were enrolled in the study. Forty five (8%) did not finished the evaluation and 67 (12%) had illegible retinography or unreliable ABP monitoring. Systolic blood pressure measured just before the retinography was 9.4 ±16.1 mmHg higher (P < 0.01) and diastolic blood pressure 26.3 ±13.9 mmHg higher (P < 0.01) than the respective 24-hours mean blood pressure.

**Table 1:** Characteristics of study population (n = 448).

	Mean ±SD or n (%)
**Age, years**	57.9 ±12.0
**Male sex, n (%)**	149 (33)
**Inner Zone**	
**Arteriolar Caliber (μm)**	105.1 ±11.3
**Venular Caliber (μm)**	129.1 ±14.6
**AV Ratio**	0.82 ±0.11
**Outer zone**	
**Arteriolar Caliber (μm)**	103.5 ±9.4
**Venular Caliber (μm)**	127.4 ±13.9
**AV Ratio**	0.82 ±0.11
**Systolic blood pressure (mmHg)**	
**At retinography**	144.6 ±23.5
**24-hours**	132.1 ±16.6
**Daytime**	135.5 ±16.7
**Nighttime**	125.2 ±18.5
**Diastolic blood pressure (mmHg)**	
**At retinography**	107.6 ±18.2
**24-hours**	77.9 ±11.8
**Daytime**	81.4 ±12.3
**Nighttime**	70.9 ±12.5

**Fig 2:**
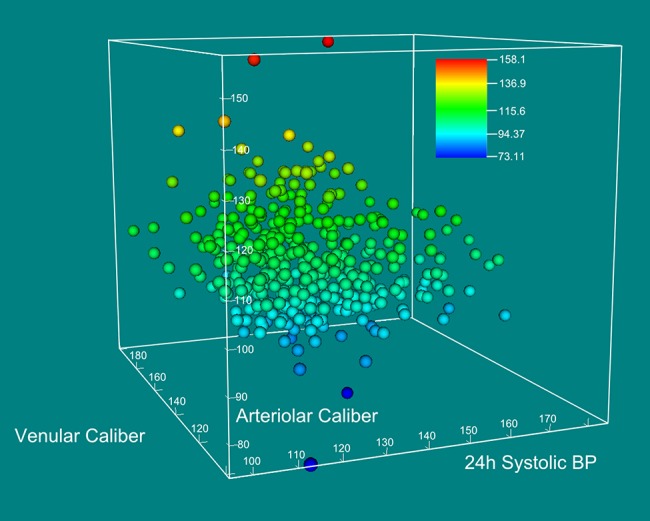
Three dimension scatter plot of arteriolar caliber (μm), 24-hours mean systolic BP (mm Hg), and venular caliber (μm). The circles were drawn at the locations specified by the vectors x, y, and z for arteriolar caliber, 24-hours systolic BP, and venular caliber.

Fig 2 shows the inverse association between arteriolar caliber and 24h systolic BP, taking into account venular caliber, in a 3-D scatter plot.

Table 2 shows that 24-hours and nightly mean systolic blood pressures were inversely associated with inner arteriolar caliber, and 24-hours and daytime mean systolic blood pressures with inner AV ratio. The 24-hours and nighttime diastolic blood pressure were inversely associated with inner and outer arteriolar caliber (approximately 1μm decrease in caliber per 10 mmHg increase of DBP, corresponding to 1% of the arteriolar caliber). Inner AV ratio was also inversely associated to 24-hours, daytime, and nighttime diastolic blood pressure, while for the outer zone, the association of AV ratio was significant for 24-hours and daytime diastolic blood pressure. Venular caliber was not associated with any of the blood pressure measurements. The arteriolar calibers decreased less and without statistical significance by increasing of systolic and diastolic blood pressures measured at the time of retinography acquisition (Table 2). The differences were higher for diastolic blood pressure, including the daytime mean BP, which was the period when the retinographies were taken.

**Table 2:** Differences in retinal vessel calibers (95% CI) per increase of 10-mmHg blood pressure, adjusted for age, fellow vessel, gender, and duration of hypertension.

**Systolic BP**	**24-hours**		**Daytime**		**Nighttime**		**At retinography**	
	Caliber (μm)	P value	Caliber (μm)	P value	Caliber (μm)	P value	Caliber (μm)	P value
**Inner zone**								
**Arteriolar**	-0.7 (-1.4 to -0.05)	0.03	-0.6 (-1.2 to 0.07)	0.08	-0.7 (-1.3 to -0.08)	0.03	-0.2 (-0.7 to 0.3)	0.4
**Venular**	0.7 (-0.2 to 1.5)	0.13	0.6 (-0.3 to 1.4)	0.17	0.5 (-0.2 to 1.3)	0.18	0.2 (-0.4 to 0.8)	0.5
**AV Ratio**	-0.01 (-0.02 to -0.001)	0.02	-0.01 (-0.02 to -0.001)	0.02	-0.01 (-0.01 to 0.001)	0.1	-0.004 (-0.01 to 0.001)	0.15
**Outer zone**								
**Arteriolar**	-0.6 (-1.2 to -0.05)	0.03	-0.6 (-1.1 to -0.01)	0.05	-0.5 (-1.0 to -0.03)	0.04	-0.4 (-0.8 to 0.7)	0.1
**Venular**	-0.1 (-0.7 to 0.9)	0.8	0.2 (-0.7 to 1.0)	0.7	-0.03 (-0.8 to 0.7)	0.9	0.2 (-0.4 to 0.9)	0.4
**AV Ratio**	-0.003 (-0.01 to 0.004)	0.4	-0.003 (-0.01 to 0.004)	0.4	-0.002 (-0.01 to 0.004)	0.5	-0.003 (-0.01 to 0.002)	0.2
**Diastolic BP**	**24-hours**		**Daytime**		**Nighttime**		**At retinography**	
	Caliber (μm)	P value	Caliber (μm)	P value	Caliber (μm)	P value	Caliber (μm)	P value
**Inner zone**								
**Arteriolar**	-1.1 (-2.0 to -0.1)	0.03	-0.9 (-1.8 to 0.04)	0.06	-1.0 (-1.9 to -0.1)	0.03	-0.2 (-0.8 to 0.5)	0.6
**Venular**	1.2 (-0.01 to 2.5)	0.05	1.1 (-0.08 to 2.3)	0.07	0.9 (-0.2 to 2.1)	0.12	0. 2 (-0.6 to 1.0)	0.6
**AV Ratio**	-0.01 (-0.02 to -0.004)	0.006	-0.01 (-0.02 to 0.004)	0.006	-0.01 (-0.02 to -0.001)	0.03	-0.003 (-0.01 to 0.004)	0.4
**Outer zone**								
**Arteriolar**	-1.2 (-2.0 to -0.4)	0.005	-1.1 (-1.9 to -0.3)	0.005	-1.0 (-1.7 to -0.2)	0.01	-0.4 (-0.9 to 0.2)	0.15
**Venular**	0.7 (-0.5 to 0.2)	0.3	0.8 (-0.3 to 2.0)	0.16	0.2 (-1.0 to 1.3)	0.8	0.4 (-0.4 to 1.2)	0.3
**AV Ratio**	-0.01 (-0.02 to -0.0001)	0.04	-0.01 (-0.02 to -0.001)	0.03	-0.01 (-0.02 to 0.004)	0.2	-0.004 (-0.01 to 0.002)	0.2

Arteriolar and venular calibers estimated by the revised Parr-Hubbard method [18,19] were approximately 50% wider than those measured by the microdensitometric method. The increase of 24h systolic BP by 10 mmHg decreased arteriolar caliber approximately in 1.9 μm (P = 0.02). The increase of 24 hours systolic blood pressure, measured at the time of retinography, accounted for smaller decrease of arteriolar caliber (1.3 μm; P = 0.04). Variation of venular caliber and AV ratio were not associated with 24h systolic BP (P value = 0.8 and 0.06, respectively). An increase of 24 hours systolic blood pressure by 10 mmHg, measured at the time of retinography, was not associated with venular caliber (P = 0.6) or AV ratio (P = 0.08). The variation of arteriolar and venular caliber and AV ratio by 10 mmHg of diastolic BP was similar to the findings observed with systolic BP, but not significant for diastolic BP measured at the time of retinography.

## Discussion

This study showed that the increasing in BP measured by ABP monitoring was associated with narrowing of retinal arteriolar caliber. The association was present for almost all periods of ABP measurements for systolic and diastolic BP. There was no association between arteriolar calibers and blood pressure measured at the time of retinography acquisition. In addition, venular calibers were not associated with increasing of blood pressure at any period of assessment. These findings suggest that structural abnormalities in the wall of retinal arterioles are more important to determine their narrowing [21] than acute variation of blood pressure.

This study is among few that demonstrated the association between blood pressure, assessed by 24-hours ABP monitoring and retinal arteriolar caliber, and it is unique to determine these parameters simultaneously. We have previously demonstrated that the diameter of the vessel measured by a microdensitometric method corresponds to the vessel lumen [10] and could be therefore influenced by vasoconstriction, endothelial damage, or even by vessel wall thickening.[22] Our findings strongly suggest that the inverse association between blood pressure and retinal vessels diameter is explained by chronic consequences of high blood pressure over retinal vessels and not by acute vasoconstriction. The absence of association between the instantaneous variation of blood pressure and arteriolar narrowing was somewhat expected. Retinal arterioles have no adrenergic supply [23] and therefore have their caliber mostly controlled by local auto regulation. Therefore, the association between retinal vessels abnormalities and chronic hypertension and its consequences are probably secondary to endothelial damage and to the reduction of the vascular lumen by arteriolar wall thickness. Abnormalities in the retinal vessels occur in parallel with abnormalities in other vascular walls, as we recently demonstrated for the carotid intima-media thickness [24].

This study confirmed the well-known inverse association between retinal arteriolar diameters and blood pressure [25–30], but just a few studies measured BP by ABP monitoring as we did [21].

In the last decade, efforts have been devoted to the evaluation of vessel calibers and AV ratio for the evaluation of hypertensive changes in the retina [31–38], some of which are widely used in clinical settings [36–38]. Several reports have expanded methodological aspects of retinal assessment. It is well known that the AV ratio overcomes the magnification differences from camera lenses and refractive error [39], even capturing less information than the numerator and denominator assessed separately [40]. In this study, the association between AV ratio and blood pressure was also confirmed for 24-hours and daytime mean systolic (inner zone) and mean diastolic ABP monitoring (inner and outer zones). Since we did find independent associations for arteriolar caliber, but not for venular caliber, AV ratio might reflect narrower arterioles in relation to venular caliber. The retinal assessment taking into account the fellow vessel might reflect the independent role of narrower arterioles. It is in disagreement with a previous study [41], that used a different set of retinal and blood pressure measurements.

In the analyses with the revised Parr-Hubbard method [19], the decrease of arteriolar caliber with the increase of BP was lower for the BP measured at the time of retinography, but maintained the significance for systolic BP. The differences among methods of retinal vessels measurement may explain these discrepancies. While the microdensitometric method measures directly all vessels in the selected zone, the Parr-Hubbard method [18] summarized the individual retinal vessel measurement into the central retinal artery or venule equivalent.

Despite the sample size and the cross-sectional design, our findings strengthen the evidence from larger cohort studies [29,30,42,43] and meta-analysis [17] linking retinal vessels calibers to high blood pressure [44]. Our study has potential limitations. Most patients had uncontrolled hypertension under drug treatment, and the influence of acute variation of blood pressure over retinal vessels at younger age and in normotensive individuals cannot be discarded. The effect of drug treatment over the constriction/dilation vessel function could eventually influence the acute response to BP variation. Nonetheless, drug treatment in this sample did not influence the association of daily and nightly BP levels. Any effect of anti-hypertensive treatment over vessels would be a conservative bias, limiting the variation of vessels calibers. Among the strengths of our study are the measurement of retinal vessels by a validated software to measure vessel calibers [3,9], the large sample size and the use of ABP monitoring.

In conclusion, the association between arteriolar retinal vessels caliber and blood pressure is not importantly influenced by instantaneous variation of blood pressure and probably depends of histological damage of arteriolar wall secondary to sustained hypertension. The evaluation of retinal vessels abnormalities by microdensitometric methods may help the risk stratification of patients with high blood pressure.
